# (*S*)-6-Chloro-4-cyclo­propyl­ethynyl-4-trifluoro­methyl-1*H*-3,1-benzoxazin-2(4*H*)-one

**DOI:** 10.1107/S1600536809049101

**Published:** 2009-11-21

**Authors:** Silvia Cuffini, R. Alan Howie, Edward R. T. Tiekink, James L. Wardell, Solange M. S. V. Wardell

**Affiliations:** a Subsecretaria Ceprocor, Ministerio de Ciência y Tecnologia de Córdoba, Alvarez de Arenales 230, 5014 Córdoba, Argentina; b Department of Chemistry, University of Aberdeen, Old Aberdeen, AB15 5NY, Scotland; c Department of Chemistry, University of Malaya, 50603 Kuala Lumpur, Malaysia; d Centro de Desenvolvimento Tecnológico em Saúde (CDTS), Fundação Oswald Cruz (FIOCRUZ), Casa Amarela, Campus de Manguinhos Av. Brasil 4365, 21040-900, Rio de Janeiro, RJ, Brazil; e CHEMSOL, 1 Harcourt Road, Aberdeen AB15 5NY, Scotland

## Abstract

Two independent mol­ecules comprise the crystallographic asymmetric unit in the title anti­retroviral agent Efavirenz, C_14_H_9_ClF_3_NO_2_, and these have noteworthy differences in conformation. The major difference relates to the orientation of the 2-cyclo­propyl­ethynyl residue relative to the six-membered heterocycle: this approaches an orthogonal disposition in mol­ecule *a* compared to a more flattened conformation in mol­ecule *b*, the difference being reflected in the O_ring_—C—C—C_ethyne_ torsion angles of 65 (4) and 159 (5)°, respectively. The independent mol­ecules are connected *via* the eight-membered {⋯HNC (O)}_2_ amide synthon. Disorder is noted in the cyclo­propane ring of mol­ecule *b* in that two orientations of equal weight were discerned.

## Related literature

For background to the use of Efavirenz, see: Adkins & Noble (1998 ▶); Gazzard (1999 ▶); de Clercq *et al.* (2009 ▶); Markowitz *et al.* (2009 ▶); Young *et al.* (2009 ▶).
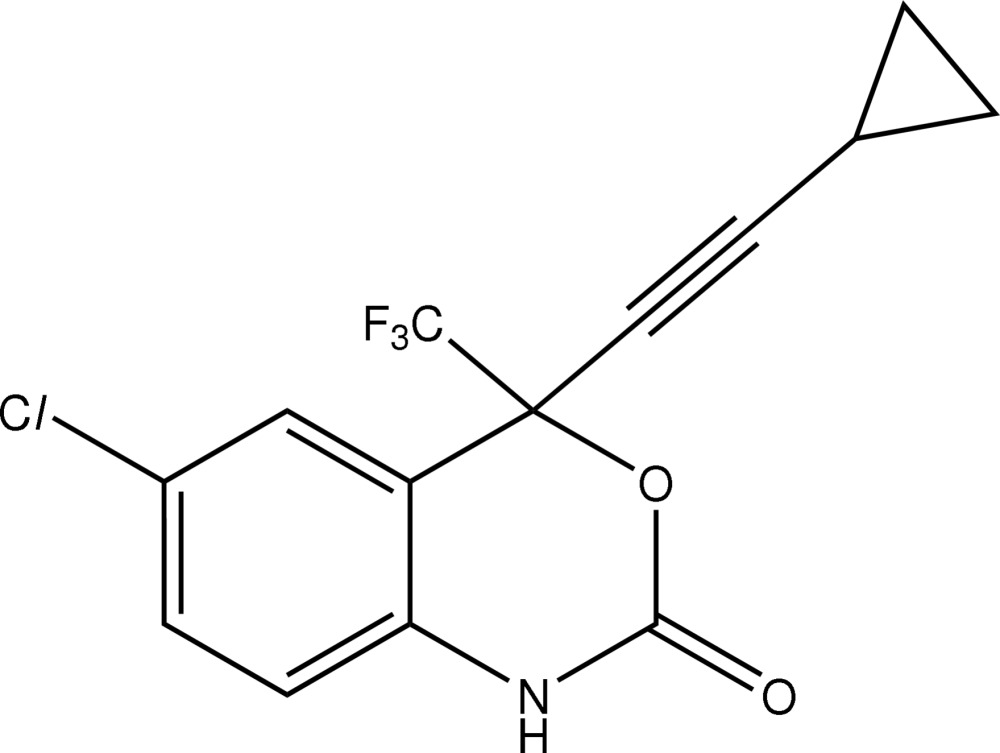


## Experimental

### 

#### Crystal data

C_14_H_9_ClF_3_NO_2_
*M* *_r_* = 315.67
Orthorhombic, 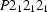

*a* = 8.1403 (4) Å
*b* = 13.5859 (11) Å
*c* = 24.962 (2) Å
*V* = 2760.6 (3) Å^3^
*Z* = 8
Mo *K*α radiation
μ = 0.31 mm^−1^
*T* = 120 K
0.28 × 0.08 × 0.04 mm

#### Data collection

Bruker–Nonius 95mm CCD camera on κ-goniostat diffractometer
Absorption correction: multi-scan (*SADABS*; Sheldrick, 2003 ▶) *T*_min_ = 0.917, *T*_max_ = 0.988
18370 measured reflections
6099 independent reflections
3675 reflections with *I* > 2σ(*I*)
*R*_int_ = 0.064

#### Refinement

*R*[*F* ^2^ > 2σ(*F* ^2^)] = 0.062
*wR*(*F* ^2^) = 0.127
*S* = 1.03
6099 reflections
395 parameters
5 restraints
H atoms treated by a mixture of independent and constrained refinement
Δρ_max_ = 0.26 e Å^−3^
Δρ_min_ = −0.25 e Å^−3^
Absolute structure: Flack (1983 ▶), 2598 Friedel pairs
Flack parameter: 0.14 (8)

### 

Data collection: *COLLECT* (Hooft, 1998 ▶); cell refinement: *DENZO* (Otwinowski & Minor, 1997 ▶) and *COLLECT*; data reduction: *DENZO* and *COLLECT*; program(s) used to solve structure: *SHELXS97* (Sheldrick, 2008 ▶); program(s) used to refine structure: *SHELXL97* (Sheldrick, 2008 ▶); molecular graphics: *DIAMOND* (Brandenburg, 2006 ▶); software used to prepare material for publication: *publCIF* (Westrip, 2009 ▶).

## Supplementary Material

Crystal structure: contains datablocks global, I. DOI: 10.1107/S1600536809049101/hg2596sup1.cif

Structure factors: contains datablocks I. DOI: 10.1107/S1600536809049101/hg2596Isup2.hkl

Additional supplementary materials:  crystallographic information; 3D view; checkCIF report

## Figures and Tables

**Table 1:** Hydrogen-bond geometry (Å, °)

*D*—H⋯*A*	*D*—H	H⋯*A*	*D*⋯*A*	*D*—H⋯*A*
N1*A*—H1*A*⋯O2*B*	0.85 (4)	2.00 (4)	2.834 (4)	167 (4)
N1*B*—H1*C*⋯O2*A*	0.89 (4)	1.94 (4)	2.820 (4)	168 (4)

**Fig. 1.**
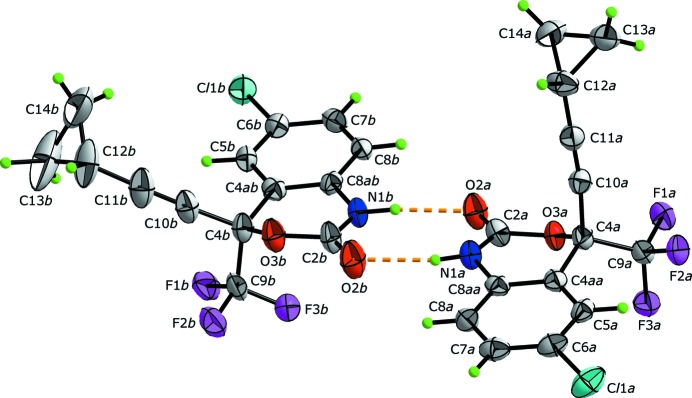
Molecular structure (I) showing the hydrogen bonding (orange dashed lines) between the two molecules comprising the crystallographic asymmetric unit, the atom-labelling scheme, and displacement ellipsoids at the 50% probability level. Only one component of the disordered C12b–C14b cyclohexane ring is shown for reasons of clarity.

## supplementary crystallographic information

### Comment

The title anti-retroviral agent, Efavirenz, is a second-generation non-nucleoside inhibitor of HIV-1 reverse transcriptase (RT) that has been approved for use against HIV-1 infection. It is also called Sustiva or Stocrin and is manufactured by Bristol-Myers Squibb. Compared with first-generation drugs such as nevirapine, Efavirenz shows greater resilience to drug resistance mutations within HIV-1 RT. Effective treatment through inhibition of HIV reverse transcriptase has been shown for both nucleoside based inhibitors, such as azidothymidine, and non-nucleoside based inhibitors, such as Efavirenz. Efavirenz is also used in combination with other anti-retroviral agents as part of an expanded post-exposure prophylaxis regimen to reduce the risk of HIV infection in people exposed to a significant risk (Adkins & Noble, 1998; Gazzard, 1999; de Clercq *et al.*, 2009; Markowitz *et al.*, 2009; Young *et al.*, 2009).

Two independent molecules comprise the asymmetric unit in (I), labelled *a* and *b*, Fig. 1. There are significant differences in conformation between the molecules and these relate primarily to the disposition of the 2-cyclopropylethynyl residue to the six-membered hetero-ring. As seen from Fig. 1, each hetero ring adopts a flattened half-chain conformation with the C4a and C4b atoms being the pivotal atoms. In molecule *a*, the C4a atom is orientated towards the same side of the six-membered ring as the 2-cyclopropylethynyl residue which occupies a position orthogonal to the six-membered ring as seen in the O3a/C4a/C10*a*/C11*a* torsion angle of 65 (4) °. In molecule *b*, the C4b atom and 2-cyclopropylethynyl residue lie to opposite sides of the six-membered ring and the comparable torsion angle is 159 (5) °. The independent molecules associate *via* the eight-membered {···HNC(O)}_2_ amide synthon, Table 1 and Fig. 1.

### Experimental

Crystals used in the crystallographic study were grown from aqueous methanol solution of (I).

### Refinement

The C-bound H atoms were geometrically placed (C–H = 0.95–1.00 Å) and refined as riding with *U_iso_*(H) = 1.2*U*_eq_(C). The amide-N H atoms were refined freely, see Table 1 for distances. Disorder was resolved for the cyclopropane ring of molecule *b* in that two positions, of equal weight (from refinement), were discerned for one of wing C atoms, C14*b*, so that each component of the disordered ring shared two atoms, C12*b* and C13*b*.

### Crystal data

**Table:**

C_14_H_9_ClF_3_NO_2_	*F*(000) = 1280
*M**_r_* = 315.67	*D*_x_ = 1.519 Mg m^−^^3^
Orthorhombic, *P*2_1_2_1_2_1_	Mo *K*α radiation, λ = 0.71073 Å
Hall symbol: P 2ac 2ab	Cell parameters from 3202 reflections
*a* = 8.1403 (4) Å	θ = 2.9–27.5°
*b* = 13.5859 (11) Å	µ = 0.31 mm^−^^1^
*c* = 24.962 (2) Å	*T* = 120 K
*V* = 2760.6 (3) Å^3^	Rod, colourless
*Z* = 8	0.28 × 0.08 × 0.04 mm

### Data collection

**Table:**

Bruker–Nonius 95mm CCD camera on κ-goniostat diffractometer	6099 independent reflections
Radiation source: Bruker-Nonius FR591 rotating anode	3675 reflections with *I* > 2σ(*I*)
10 cm confocal mirrors	*R*_int_ = 0.064
Detector resolution: 9.091 pixels mm^-1^	θ_max_ = 27.5°, θ_min_ = 2.9°
φ and ω scans	*h* = −10→6
Absorption correction: multi-scan (*SADABS*; Sheldrick, 2003)	*k* = −10→17
*T*_min_ = 0.917, *T*_max_ = 0.988	*l* = −32→30
18370 measured reflections	

### Refinement

**Table:**

Refinement on *F*^2^	Secondary atom site location: difference Fourier map
Least-squares matrix: full	Hydrogen site location: inferred from neighbouring sites
*R*[*F*^2^ > 2σ(*F*^2^)] = 0.062	H atoms treated by a mixture of independent and constrained refinement
*wR*(*F*^2^) = 0.127	*w* = 1/[σ^2^(*F*_o_^2^) + (0.0502*P*)^2^ + 0.0117*P*] where *P* = (*F*_o_^2^ + 2*F*_c_^2^)/3
*S* = 1.03	(Δ/σ)_max_ = 0.001
6099 reflections	Δρ_max_ = 0.26 e Å^−^^3^
395 parameters	Δρ_min_ = −0.25 e Å^−^^3^
5 restraints	Absolute structure: Flack (1983), 2598 Friedel pairs
Primary atom site location: structure-invariant direct methods	Flack parameter: 0.14 (8)

### Special details

**Table:**

Geometry. All s.u.'s (except the s.u. in the dihedral angle between two l.s. planes) are estimated using the full covariance matrix. The cell s.u.'s are taken into account individually in the estimation of s.u.'s in distances, angles and torsion angles; correlations between s.u.'s in cell parameters are only used when they are defined by crystal symmetry. An approximate (isotropic) treatment of cell s.u.'s is used for estimating s.u.'s involving l.s. planes.
Refinement. Refinement of *F*^2^ against ALL reflections. The weighted *R*-factor *wR* and goodness of fit *S* are based on *F*^2^, conventional *R*-factors *R* are based on *F*, with *F* set to zero for negative *F*^2^. The threshold expression of *F*^2^ > 2σ(*F*^2^) is used only for calculating *R*-factors(gt) *etc*. and is not relevant to the choice of reflections for refinement. *R*-factors based on *F*^2^ are statistically about twice as large as those based on *F*, and *R*- factors based on ALL data will be even larger.

### Fractional atomic coordinates and isotropic or equivalent isotropic displacement parameters (Å^2^)

**Table:**

	*x*	*y*	*z*	*U*_iso_*/*U*_eq_	Occ. (<1)
Cl1A	−0.51005 (11)	0.32715 (8)	0.73151 (5)	0.0602 (3)	
O2A	0.3501 (3)	0.5959 (2)	0.74717 (10)	0.0507 (8)	
O3A	0.1228 (3)	0.59292 (19)	0.69880 (10)	0.0370 (6)	
N1A	0.1615 (4)	0.4768 (2)	0.76479 (13)	0.0358 (8)	
H1A	0.207 (5)	0.457 (3)	0.7936 (15)	0.043*	
F1A	−0.0025 (3)	0.65810 (18)	0.60948 (9)	0.0526 (6)	
F2A	−0.1987 (3)	0.55204 (18)	0.60690 (8)	0.0512 (6)	
F3A	−0.1848 (3)	0.65650 (16)	0.67151 (8)	0.0497 (6)	
C2A	0.2190 (5)	0.5547 (3)	0.73799 (15)	0.0377 (9)	
C4A	0.0094 (5)	0.5259 (3)	0.67159 (14)	0.0348 (9)	
C4AA	−0.0859 (4)	0.4654 (3)	0.71220 (14)	0.0334 (9)	
C5A	−0.2433 (4)	0.4314 (3)	0.70474 (15)	0.0343 (9)	
H5A	−0.3042	0.4503	0.6739	0.045*	
C6A	−0.3118 (4)	0.3698 (3)	0.74239 (17)	0.0434 (10)	
C7A	−0.2254 (4)	0.3397 (3)	0.78715 (16)	0.0426 (10)	
H7A	−0.2726	0.2948	0.8119	0.055*	
C8A	−0.0678 (4)	0.3764 (3)	0.79520 (16)	0.0369 (9)	
H8A	−0.0077	0.3583	0.8263	0.048*	
C8AA	0.0015 (4)	0.4393 (3)	0.75789 (14)	0.0326 (9)	
C9A	−0.0952 (5)	0.5989 (3)	0.63969 (15)	0.0398 (10)	
C10A	0.1021 (4)	0.4604 (3)	0.63575 (16)	0.0366 (9)	
C11A	0.1844 (5)	0.4037 (3)	0.61164 (16)	0.0411 (10)	
C12A	0.2841 (5)	0.3343 (3)	0.58197 (16)	0.0507 (11)	
H12A	0.2696	0.2635	0.5919	0.061*	
C13A	0.3236 (5)	0.3538 (3)	0.52437 (17)	0.0530 (12)	
H13A	0.3292	0.2967	0.4998	0.064*	
H13B	0.2802	0.4150	0.5081	0.064*	
C14A	0.4533 (5)	0.3634 (4)	0.56533 (18)	0.0597 (13)	
H14A	0.4909	0.4306	0.5746	0.072*	
H14B	0.5400	0.3123	0.5663	0.072*	
Cl1B	1.21317 (12)	0.70229 (9)	0.86981 (4)	0.0532 (3)	
O2B	0.3568 (3)	0.4274 (2)	0.85483 (11)	0.0607 (9)	
O3B	0.5822 (3)	0.4297 (2)	0.90321 (11)	0.0470 (8)	
N1B	0.5495 (4)	0.5424 (3)	0.83487 (13)	0.0392 (9)	
H1C	0.498 (5)	0.566 (3)	0.8061 (15)	0.047*	
F1B	0.7286 (3)	0.60158 (18)	1.00057 (9)	0.0534 (6)	
F2B	0.5402 (3)	0.49001 (19)	1.00565 (9)	0.0612 (7)	
F3B	0.5136 (3)	0.61081 (19)	0.95046 (9)	0.0539 (6)	
C2B	0.4865 (5)	0.4670 (3)	0.86305 (16)	0.0445 (10)	
C4B	0.7093 (5)	0.4883 (3)	0.92846 (15)	0.0388 (9)	
C4BA	0.7914 (4)	0.5559 (3)	0.88837 (14)	0.0333 (9)	
C5B	0.9506 (4)	0.5922 (3)	0.89599 (15)	0.0360 (9)	
H5C	1.0124	0.5729	0.9265	0.047*	
C6B	1.0174 (4)	0.6561 (3)	0.85898 (15)	0.0375 (9)	
C7B	0.9312 (4)	0.6826 (3)	0.81354 (15)	0.0372 (10)	
H7C	0.9780	0.7271	0.7885	0.048*	
C8B	0.7766 (4)	0.6439 (3)	0.80484 (15)	0.0356 (9)	
H8C	0.7182	0.6598	0.7731	0.046*	
C8BA	0.7071 (4)	0.5820 (3)	0.84245 (15)	0.0344 (9)	
C9B	0.6220 (5)	0.5474 (3)	0.97213 (15)	0.0440 (11)	
C10B	0.8249 (5)	0.4199 (3)	0.95367 (16)	0.0461 (11)	
C11B	0.9272 (6)	0.3707 (3)	0.97389 (19)	0.0599 (13)	
C12B	1.0542 (6)	0.3081 (4)	0.9974 (2)	0.0782 (17)	
H12C	1.0219	0.2386	1.0052	0.094*	0.50
H12E	1.0798	0.2445	0.9791	0.094*	0.50
C13B	1.1770 (9)	0.3495 (4)	1.0313 (4)	0.120 (3)	
H13C	1.2225	0.3075	1.0601	0.144*	0.50
H13D	1.1678	0.4202	1.0404	0.144*	0.50
H13E	1.1876	0.4221	1.0312	0.144*	0.50
H13F	1.2834	0.3143	1.0332	0.144*	0.50
C14B	1.2189 (13)	0.3225 (12)	0.9731 (6)	0.135 (6)	0.50
H14C	1.2329	0.3767	0.9470	0.162*	0.50
H14D	1.2878	0.2637	0.9667	0.162*	0.50
C14C	1.0387 (19)	0.3074 (14)	1.0626 (5)	0.165 (9)	0.50
H14E	0.9600	0.3532	1.0796	0.198*	0.50
H14F	1.0567	0.2444	1.0817	0.198*	0.50

### Atomic displacement parameters (Å^2^)

**Table:**

	*U*^11^	*U*^22^	*U*^33^	*U*^12^	*U*^13^	*U*^23^
Cl1A	0.0346 (5)	0.0526 (7)	0.0935 (9)	−0.0044 (5)	0.0009 (5)	0.0029 (7)
O2A	0.0581 (18)	0.0487 (19)	0.0454 (18)	−0.0176 (14)	−0.0172 (14)	0.0066 (15)
O3A	0.0439 (15)	0.0350 (17)	0.0321 (15)	−0.0036 (12)	−0.0100 (12)	0.0013 (13)
N1A	0.0408 (19)	0.034 (2)	0.033 (2)	−0.0030 (14)	−0.0090 (14)	0.0037 (17)
F1A	0.0536 (13)	0.0630 (16)	0.0411 (14)	0.0041 (13)	0.0026 (11)	0.0163 (13)
F2A	0.0464 (13)	0.0691 (17)	0.0380 (13)	0.0053 (12)	−0.0114 (11)	−0.0007 (13)
F3A	0.0596 (14)	0.0504 (15)	0.0393 (13)	0.0158 (12)	0.0032 (10)	0.0000 (12)
C2A	0.046 (2)	0.037 (2)	0.031 (2)	−0.004 (2)	−0.0032 (19)	0.001 (2)
C4A	0.036 (2)	0.039 (2)	0.029 (2)	0.0024 (18)	−0.0054 (16)	−0.0042 (19)
C4AA	0.040 (2)	0.031 (2)	0.030 (2)	0.0059 (17)	0.0014 (16)	−0.0010 (19)
C5A	0.031 (2)	0.034 (2)	0.037 (2)	0.0050 (16)	−0.0057 (16)	−0.0082 (19)
C6A	0.039 (2)	0.036 (3)	0.055 (3)	0.0028 (18)	0.005 (2)	−0.006 (2)
C7A	0.045 (2)	0.033 (2)	0.049 (3)	0.0018 (19)	0.011 (2)	−0.003 (2)
C8A	0.044 (2)	0.032 (2)	0.035 (2)	0.0074 (17)	0.0016 (18)	0.0007 (19)
C8AA	0.036 (2)	0.031 (2)	0.030 (2)	0.0026 (17)	0.0033 (17)	−0.0059 (18)
C9A	0.046 (2)	0.044 (3)	0.030 (2)	0.008 (2)	0.0004 (18)	0.002 (2)
C10A	0.034 (2)	0.041 (3)	0.034 (2)	−0.0009 (18)	−0.0027 (17)	−0.001 (2)
C11A	0.040 (2)	0.044 (3)	0.039 (2)	−0.001 (2)	−0.0028 (19)	0.005 (2)
C12A	0.061 (3)	0.040 (3)	0.051 (3)	0.002 (2)	0.018 (2)	0.005 (2)
C13A	0.054 (3)	0.055 (3)	0.049 (3)	0.005 (2)	0.012 (2)	−0.001 (2)
C14A	0.042 (3)	0.068 (4)	0.070 (3)	0.012 (2)	0.007 (2)	0.003 (3)
Cl1B	0.0405 (6)	0.0650 (8)	0.0541 (7)	−0.0059 (5)	−0.0014 (5)	0.0164 (6)
O2B	0.0549 (19)	0.073 (2)	0.054 (2)	−0.0245 (17)	−0.0208 (15)	0.0135 (17)
O3B	0.0509 (16)	0.0488 (19)	0.0415 (18)	−0.0135 (14)	−0.0137 (13)	0.0082 (15)
N1B	0.045 (2)	0.043 (2)	0.029 (2)	0.0009 (15)	−0.0112 (14)	0.0047 (18)
F1B	0.0537 (14)	0.0714 (17)	0.0350 (12)	−0.0047 (12)	−0.0044 (11)	−0.0097 (13)
F2B	0.0564 (15)	0.088 (2)	0.0389 (15)	−0.0101 (13)	0.0021 (11)	0.0188 (14)
F3B	0.0457 (13)	0.0757 (18)	0.0402 (14)	0.0112 (13)	−0.0009 (11)	0.0075 (13)
C2B	0.048 (3)	0.052 (3)	0.034 (2)	−0.006 (2)	−0.016 (2)	0.004 (2)
C4B	0.039 (2)	0.040 (2)	0.037 (2)	−0.0039 (19)	−0.0116 (18)	0.002 (2)
C4BA	0.038 (2)	0.031 (2)	0.031 (2)	0.0003 (18)	0.0004 (17)	−0.0039 (18)
C5B	0.037 (2)	0.039 (3)	0.032 (2)	0.0036 (17)	−0.0053 (17)	0.006 (2)
C6B	0.034 (2)	0.041 (2)	0.037 (2)	0.0032 (18)	−0.0011 (17)	0.003 (2)
C7B	0.048 (2)	0.032 (2)	0.032 (2)	0.0035 (19)	0.0070 (17)	0.0009 (19)
C8B	0.046 (2)	0.034 (2)	0.026 (2)	0.0097 (18)	0.0012 (17)	0.0017 (18)
C8BA	0.032 (2)	0.038 (2)	0.033 (2)	0.0078 (17)	−0.0056 (17)	−0.0085 (19)
C9B	0.045 (3)	0.062 (3)	0.025 (2)	−0.006 (2)	−0.0056 (19)	0.010 (2)
C10B	0.052 (3)	0.046 (3)	0.040 (3)	−0.012 (2)	−0.010 (2)	0.011 (2)
C11B	0.066 (3)	0.049 (3)	0.064 (3)	−0.012 (2)	−0.022 (3)	0.016 (3)
C12B	0.094 (4)	0.044 (3)	0.096 (4)	0.002 (3)	−0.042 (3)	0.022 (3)
C13B	0.129 (6)	0.061 (4)	0.169 (8)	0.032 (4)	−0.099 (6)	−0.027 (5)
C14B	0.071 (8)	0.197 (17)	0.136 (13)	0.054 (9)	0.004 (8)	0.086 (13)
C14C	0.169 (15)	0.24 (2)	0.081 (10)	0.143 (16)	0.016 (9)	0.061 (12)

### Geometric parameters (Å, °)

**Table:**

Cl1A—C6A	1.736 (4)	N1B—C2B	1.344 (5)
O2A—C2A	1.227 (4)	N1B—C8BA	1.404 (5)
O3A—C2A	1.356 (4)	N1B—H1C	0.89 (4)
O3A—C4A	1.464 (4)	F1B—C9B	1.341 (4)
N1A—C2A	1.337 (5)	F2B—C9B	1.323 (4)
N1A—C8AA	1.409 (5)	F3B—C9B	1.346 (4)
N1A—H1A	0.85 (4)	C4B—C10B	1.465 (5)
F1A—C9A	1.336 (4)	C4B—C4BA	1.514 (5)
F2A—C9A	1.336 (4)	C4B—C9B	1.529 (6)
F3A—C9A	1.333 (4)	C4BA—C8BA	1.383 (5)
C4A—C10A	1.470 (6)	C4BA—C5B	1.400 (5)
C4A—C4AA	1.518 (5)	C5B—C6B	1.379 (5)
C4A—C9A	1.531 (5)	C5B—H5C	0.9500
C4AA—C5A	1.375 (5)	C6B—C7B	1.381 (5)
C4AA—C8AA	1.390 (5)	C7B—C8B	1.381 (5)
C5A—C6A	1.377 (5)	C7B—H7C	0.9500
C5A—H5A	0.9500	C8B—C8BA	1.381 (5)
C6A—C7A	1.382 (5)	C8B—H8C	0.9500
C7A—C8A	1.391 (5)	C10B—C11B	1.181 (5)
C7A—H7A	0.9500	C11B—C12B	1.461 (6)
C8A—C8AA	1.384 (5)	C12B—C13B	1.425 (7)
C8A—H8A	0.9500	C12B—C14B	1.484 (11)
C10A—C11A	1.186 (5)	C12B—C14C	1.632 (12)
C11A—C12A	1.448 (6)	C12B—H12C	1.0000
C12A—C14A	1.492 (5)	C12B—H12E	1.0000
C12A—C13A	1.497 (5)	C13B—C14C	1.485 (14)
C12A—H12A	1.0000	C13B—C14B	1.536 (13)
C13A—C14A	1.475 (6)	C13B—H13C	0.9900
C13A—H13A	0.9900	C13B—H13D	0.9900
C13A—H13B	0.9900	C13B—H13E	0.9900
C14A—H14A	0.9900	C13B—H13F	0.9900
C14A—H14B	0.9900	C14B—H14C	0.9900
Cl1B—C6B	1.734 (4)	C14B—H14D	0.9900
O2B—C2B	1.202 (5)	C14C—H14E	0.9900
O3B—C2B	1.368 (4)	C14C—H14F	0.9900
O3B—C4B	1.450 (4)		
			
C2A—O3A—C4A	117.4 (3)	C5B—C4BA—C4B	122.2 (3)
C2A—N1A—C8AA	123.3 (3)	C6B—C5B—C4BA	119.7 (3)
C2A—N1A—H1A	121 (3)	C6B—C5B—H5C	120.1
C8AA—N1A—H1A	113 (3)	C4BA—C5B—H5C	120.1
O2A—C2A—N1A	124.9 (4)	C5B—C6B—C7B	120.9 (3)
O2A—C2A—O3A	117.6 (3)	C5B—C6B—Cl1B	119.1 (3)
N1A—C2A—O3A	117.5 (3)	C7B—C6B—Cl1B	120.0 (3)
O3A—C4A—C10A	109.6 (3)	C8B—C7B—C6B	119.5 (4)
O3A—C4A—C4AA	110.4 (3)	C8B—C7B—H7C	120.2
C10A—C4A—C4AA	110.0 (3)	C6B—C7B—H7C	120.2
O3A—C4A—C9A	100.9 (3)	C7B—C8B—C8BA	119.9 (3)
C10A—C4A—C9A	111.1 (3)	C7B—C8B—H8C	120.1
C4AA—C4A—C9A	114.4 (3)	C8BA—C8B—H8C	120.1
C5A—C4AA—C8AA	120.1 (3)	C8B—C8BA—C4BA	121.1 (3)
C5A—C4AA—C4A	124.6 (3)	C8B—C8BA—N1B	121.1 (3)
C8AA—C4AA—C4A	115.1 (3)	C4BA—C8BA—N1B	117.8 (3)
C4AA—C5A—C6A	119.3 (3)	F2B—C9B—F1B	108.3 (3)
C4AA—C5A—H5A	120.3	F2B—C9B—F3B	107.5 (3)
C6A—C5A—H5A	120.3	F1B—C9B—F3B	106.6 (4)
C5A—C6A—C7A	121.7 (4)	F2B—C9B—C4B	112.0 (4)
C5A—C6A—Cl1A	118.2 (3)	F1B—C9B—C4B	111.4 (3)
C7A—C6A—Cl1A	120.1 (3)	F3B—C9B—C4B	110.8 (3)
C6A—C7A—C8A	118.7 (4)	C11B—C10B—C4B	174.7 (4)
C6A—C7A—H7A	120.6	C10B—C11B—C12B	178.3 (6)
C8A—C7A—H7A	120.6	C13B—C12B—C11B	120.4 (5)
C8AA—C8A—C7A	120.0 (4)	C13B—C12B—C14B	63.7 (6)
C8AA—C8A—H8A	120.0	C11B—C12B—C14B	113.4 (6)
C7A—C8A—H8A	120.0	C13B—C12B—C14C	57.7 (6)
C8A—C8AA—C4AA	120.1 (4)	C11B—C12B—C14C	110.5 (7)
C8A—C8AA—N1A	121.2 (3)	C14B—C12B—C14C	118.6 (8)
C4AA—C8AA—N1A	118.7 (3)	C13B—C12B—H12C	116.2
F3A—C9A—F1A	107.0 (3)	C11B—C12B—H12C	116.2
F3A—C9A—F2A	107.4 (3)	C14B—C12B—H12C	116.2
F1A—C9A—F2A	107.3 (3)	C14C—C12B—H12C	77.3
F3A—C9A—C4A	112.0 (3)	C13B—C12B—H12E	117.8
F1A—C9A—C4A	111.7 (3)	C11B—C12B—H12E	117.8
F2A—C9A—C4A	111.2 (3)	C14B—C12B—H12E	74.9
C11A—C10A—C4A	173.0 (4)	C14C—C12B—H12E	117.8
C10A—C11A—C12A	179.7 (5)	H12C—C12B—H12E	47.7
C11A—C12A—C14A	119.2 (4)	C12B—C13B—C14C	68.2 (6)
C11A—C12A—C13A	119.8 (4)	C12B—C13B—C14B	60.0 (5)
C14A—C12A—C13A	59.2 (3)	C14C—C13B—C14B	125.0 (9)
C11A—C12A—H12A	115.7	C12B—C13B—H13C	117.8
C14A—C12A—H12A	115.7	C14C—C13B—H13C	71.3
C13A—C12A—H12A	115.7	C14B—C13B—H13C	117.8
C14A—C13A—C12A	60.3 (3)	C12B—C13B—H13D	117.8
C14A—C13A—H13A	117.7	C14C—C13B—H13D	101.3
C12A—C13A—H13A	117.7	C14B—C13B—H13D	117.8
C14A—C13A—H13B	117.7	H13C—C13B—H13D	114.9
C12A—C13A—H13B	117.7	C12B—C13B—H13E	116.9
H13A—C13A—H13B	114.9	C14C—C13B—H13E	116.9
C13A—C14A—C12A	60.6 (3)	C14B—C13B—H13E	102.4
C13A—C14A—H14A	117.7	H13C—C13B—H13E	123.0
C12A—C14A—H14A	117.7	C12B—C13B—H13F	116.9
C13A—C14A—H14B	117.7	C14C—C13B—H13F	116.9
C12A—C14A—H14B	117.7	C14B—C13B—H13F	74.7
H14A—C14A—H14B	114.8	H13C—C13B—H13F	50.2
C2B—O3B—C4B	121.4 (3)	H13D—C13B—H13F	121.6
C2B—N1B—C8BA	124.8 (4)	H13E—C13B—H13F	113.9
C2B—N1B—H1C	121 (3)	C12B—C14B—C13B	56.3 (5)
C8BA—N1B—H1C	114 (3)	C12B—C14B—H14C	118.1
O2B—C2B—N1B	126.0 (4)	C13B—C14B—H14C	118.1
O2B—C2B—O3B	117.3 (4)	C12B—C14B—H14D	118.1
N1B—C2B—O3B	116.7 (4)	C13B—C14B—H14D	118.1
O3B—C4B—C10B	107.3 (3)	H14C—C14B—H14D	115.3
O3B—C4B—C4BA	111.2 (3)	C13B—C14C—C12B	54.2 (5)
C10B—C4B—C4BA	112.6 (3)	C13B—C14C—H14E	118.3
O3B—C4B—C9B	105.5 (3)	C12B—C14C—H14E	118.3
C10B—C4B—C9B	109.0 (3)	C13B—C14C—H14F	118.3
C4BA—C4B—C9B	111.0 (3)	C12B—C14C—H14F	118.3
C8BA—C4BA—C5B	118.8 (3)	H14E—C14C—H14F	115.6
C8BA—C4BA—C4B	119.0 (3)		
			
C8AA—N1A—C2A—O2A	171.5 (4)	C2B—O3B—C4B—C4BA	−35.7 (5)
C8AA—N1A—C2A—O3A	−6.4 (5)	C2B—O3B—C4B—C9B	84.6 (4)
C4A—O3A—C2A—O2A	153.8 (3)	O3B—C4B—C4BA—C8BA	23.4 (5)
C4A—O3A—C2A—N1A	−28.2 (5)	C10B—C4B—C4BA—C8BA	143.8 (3)
C2A—O3A—C4A—C10A	−73.1 (4)	C9B—C4B—C4BA—C8BA	−93.6 (4)
C2A—O3A—C4A—C4AA	48.2 (4)	O3B—C4B—C4BA—C5B	−156.0 (3)
C2A—O3A—C4A—C9A	169.6 (3)	C10B—C4B—C4BA—C5B	−35.6 (5)
O3A—C4A—C4AA—C5A	149.0 (3)	C9B—C4B—C4BA—C5B	86.9 (4)
C10A—C4A—C4AA—C5A	−89.9 (4)	C8BA—C4BA—C5B—C6B	2.6 (5)
C9A—C4A—C4AA—C5A	36.0 (5)	C4B—C4BA—C5B—C6B	−177.9 (3)
O3A—C4A—C4AA—C8AA	−35.3 (4)	C4BA—C5B—C6B—C7B	−1.9 (6)
C10A—C4A—C4AA—C8AA	85.8 (4)	C4BA—C5B—C6B—Cl1B	178.7 (3)
C9A—C4A—C4AA—C8AA	−148.3 (3)	C5B—C6B—C7B—C8B	−0.6 (6)
C8AA—C4AA—C5A—C6A	−1.3 (5)	Cl1B—C6B—C7B—C8B	178.8 (3)
C4A—C4AA—C5A—C6A	174.1 (3)	C6B—C7B—C8B—C8BA	2.3 (5)
C4AA—C5A—C6A—C7A	−1.1 (6)	C7B—C8B—C8BA—C4BA	−1.6 (5)
C4AA—C5A—C6A—Cl1A	−179.4 (3)	C7B—C8B—C8BA—N1B	179.4 (3)
C5A—C6A—C7A—C8A	2.8 (6)	C5B—C4BA—C8BA—C8B	−0.8 (5)
Cl1A—C6A—C7A—C8A	−179.0 (3)	C4B—C4BA—C8BA—C8B	179.7 (3)
C6A—C7A—C8A—C8AA	−2.1 (5)	C5B—C4BA—C8BA—N1B	178.2 (3)
C7A—C8A—C8AA—C4AA	−0.3 (6)	C4B—C4BA—C8BA—N1B	−1.3 (5)
C7A—C8A—C8AA—N1A	−179.4 (3)	C2B—N1B—C8BA—C8B	165.5 (4)
C5A—C4AA—C8AA—C8A	2.0 (5)	C2B—N1B—C8BA—C4BA	−13.5 (5)
C4A—C4AA—C8AA—C8A	−173.8 (3)	O3B—C4B—C9B—F2B	55.3 (4)
C5A—C4AA—C8AA—N1A	−178.9 (3)	C10B—C4B—C9B—F2B	−59.6 (4)
C4A—C4AA—C8AA—N1A	5.3 (5)	C4BA—C4B—C9B—F2B	175.8 (3)
C2A—N1A—C8AA—C8A	−163.0 (4)	O3B—C4B—C9B—F1B	176.8 (3)
C2A—N1A—C8AA—C4AA	17.9 (5)	C10B—C4B—C9B—F1B	61.9 (4)
O3A—C4A—C9A—F3A	−66.2 (4)	C4BA—C4B—C9B—F1B	−62.7 (4)
C10A—C4A—C9A—F3A	177.7 (3)	O3B—C4B—C9B—F3B	−64.7 (4)
C4AA—C4A—C9A—F3A	52.4 (4)	C10B—C4B—C9B—F3B	−179.6 (3)
O3A—C4A—C9A—F1A	53.8 (4)	C4BA—C4B—C9B—F3B	55.8 (4)
C10A—C4A—C9A—F1A	−62.3 (4)	O3B—C4B—C10B—C11B	159 (5)
C4AA—C4A—C9A—F1A	172.4 (3)	C4BA—C4B—C10B—C11B	36 (5)
O3A—C4A—C9A—F2A	173.6 (3)	C9B—C4B—C10B—C11B	−88 (5)
C10A—C4A—C9A—F2A	57.5 (4)	C4B—C10B—C11B—C12B	−107 (17)
C4AA—C4A—C9A—F2A	−67.8 (4)	C10B—C11B—C12B—C13B	140 (18)
O3A—C4A—C10A—C11A	65 (4)	C10B—C11B—C12B—C14B	67 (18)
C4AA—C4A—C10A—C11A	−57 (4)	C10B—C11B—C12B—C14C	−157 (18)
C9A—C4A—C10A—C11A	176 (3)	C11B—C12B—C13B—C14C	96.3 (9)
C4A—C10A—C11A—C12A	147 (100)	C14B—C12B—C13B—C14C	−160.8 (10)
C10A—C11A—C12A—C14A	125 (100)	C11B—C12B—C13B—C14B	−103.0 (8)
C10A—C11A—C12A—C13A	56 (97)	C14C—C12B—C13B—C14B	160.8 (10)
C11A—C12A—C13A—C14A	108.2 (5)	C11B—C12B—C14B—C13B	113.6 (6)
C11A—C12A—C14A—C13A	−109.2 (4)	C14C—C12B—C14B—C13B	−18.5 (10)
C8BA—N1B—C2B—O2B	−174.4 (4)	C14C—C13B—C14B—C12B	21.9 (10)
C8BA—N1B—C2B—O3B	2.6 (6)	C14B—C13B—C14C—C12B	−20.4 (9)
C4B—O3B—C2B—O2B	−158.9 (4)	C11B—C12B—C14C—C13B	−113.7 (6)
C4B—O3B—C2B—N1B	23.9 (5)	C14B—C12B—C14C—C13B	19.7 (10)
C2B—O3B—C4B—C10B	−159.3 (3)		

### Hydrogen-bond geometry (Å, °)

**Table:**

*D*—H···*A*	*D*—H	H···*A*	*D*···*A*	*D*—H···*A*
N1A—H1A···O2B	0.85 (4)	2.00 (4)	2.834 (4)	167 (4)
N1B—H1C···O2A	0.89 (4)	1.94 (4)	2.820 (4)	168 (4)
